# Test–retest reliability of arterial spin labelling for cerebral blood flow in older adults with small vessel disease

**DOI:** 10.1007/s12975-021-00983-5

**Published:** 2022-01-26

**Authors:** Lauren R. Binnie, Mathilde M. H. Pauls, Philip Benjamin, Mohani-Preet K. Dhillon, Shai Betteridge, Brian Clarke, Rita Ghatala, Fearghal A. H. Hainsworth, Franklyn A. Howe, Usman Khan, Christina Kruuse, Jeremy B. Madigan, Barry Moynihan, Bhavini Patel, Anthony C. Pereira, Egill Rostrup, Anan B. Y. Shtaya, Catherine A. Spilling, Sarah Trippier, Rebecca Williams, Jeremy D. Isaacs, Thomas R. Barrick, Atticus H. Hainsworth

**Affiliations:** 1 Present Address: Molecular & Clinical Sciences Research Institute, St George’s University of London, Cranmer Terrace, London, SW17 0RE UK; 2 Department of Neurology, St George’s University Hospitals NHS Foundation Trust London, London, UK; 3 Department of Neuroradiology, St George’s University Hospitals NHS Foundation Trust London, London, UK; 4 Department of Neuropsychology, St George’s University Hospitals NHS Foundation Trust London, London, UK; 5 Department of Neurology and Neurovascular Research Unit, Herlev Gentofte Hospital, Herlev, Denmark; 6 Department of Medicine, Royal College of Surgeons in Ireland, Beaumont Hospital, Dublin, Ireland; 7 Mental Health Centre, University of Copenhagen, Glostrup, Denmark; 8 Present Address: South London Stroke Research Network, St George’s Hospital, London, UK

**Keywords:** Arterial spin labelling, Cerebral blood flow, Small vessel disease, Vascular aging, White matter lesions

## Abstract

Cerebral small vessel disease (SVD) is common in older people and is associated with lacunar stroke, white matter hyperintensities (WMH) and vascular cognitive impairment. Cerebral blood flow (CBF) is reduced in SVD, particularly within white matter.

Here we quantified test–retest reliability in CBF measurements using pseudo-continuous arterial spin labelling (pCASL) in older adults with clinical and radiological evidence of SVD (N=54, mean (SD): 66.9 (8.7) years, 15 females/39 males). We generated whole-brain CBF maps on two visits at least 7 days apart (mean (SD): 20 (19), range 7-117 days).

Test–retest reliability for CBF was high in all tissue types, with intra-class correlation coefficient [95%CI]: 0.758 [0.616, 0.852] for whole brain, 0.842 [0.743, 0.905] for total grey matter, 0.771 [0.636, 0.861] for deep grey matter (caudate-putamen and thalamus), 0.872 [0.790, 0.923] for normal-appearing white matter (NAWM) and 0.780 [0.650, 0.866] for WMH (all p<0.001). ANCOVA models indicated significant decline in CBF in total grey matter, deep grey matter and NAWM with increasing age and diastolic blood pressure (all p<0.001). CBF was lower in males relative to females (p=0.013 for total grey matter, p=0.004 for NAWM).

We conclude that pCASL has high test–retest reliability as a quantitative measure of CBF in older adults with SVD. These findings support the use of pCASL in routine clinical imaging and as a clinical trial endpoint.

All data come from the PASTIS trial, prospectively registered at: https://eudract.ema.europa.eu (2015-001235-20, registered 13/05/2015), http://www.clinicaltrials.gov (NCT02450253, registered 21/05/2015).

**Supplementary Information:**

The online version contains supplementary material available at 10.1007/s12975-021-00983-5.

## Introduction

Cerebral small vessel disease (SVD) is common in older people, observed radiologically as focal lacunar infarcts, micro-haemorrhages and diffuse white matter hyperintensities (WMH) on T2-weighted MRI scans [1, 2]. SVD is the main cause of clinical lacunar stroke and a major source of vascular contributions to cognitive impairment and dementia (VCID) [3–6]. SVD is associated with reduced cerebral blood flow (CBF) [7, 8]. Resting CBF is lower in individuals with SVD compared to healthy aged control subjects, particularly in subcortical white matter [7–14].

Arterial spin labelling is now well developed as an MRI-derived quantitative measure of CBF. As the test–retest reproducibility of this method has not been quantified in people with SVD, it has not entered routine use for clinical assessment or as a clinical trial outcome measure. Here we report whole-brain CBF maps measured by pseudo-continuous arterial spin labelling (pCASL) [15, 16] in a well-characterised cohort of older adults with symptomatic SVD [17]. First, we aimed to test whether pCASL is an effective method for quantitative assessment of CBF in people with SVD, particularly in white matter areas where absolute CBF values are low [7, 10, 18]. Second, we aimed to quantify test–retest reliability in CBF measured by pCASL within this participant group, using CBF maps derived from two successive visits, at least 7 days apart.

## Methods

### Study population

All data are from a cohort of older adults with radiological and clinical evidence of symptomatic SVD (N=54, demographic details in Table 1). These participants were all recruited as part of a double-blinded, placebo-controlled, phase-II clinical trial, Perfusion by Arterial spin labelling following Single dose Tadalafil In Small vessel disease (PASTIS; European Union Clinical Trials Register number 2015-001235-20, registered 13/05/2015) [17]. The trial received ethical approval from the UK National Research Ethics Service (REC reference: 15/LO/0714). Further details are given in the Supplementary file.

**Table 1:** Participant demographics for the study cohort

Variable	All	Female	Male	P-value
N	54	15	39	
Age in years	66.9 (8.7)	69.9 (8.65)	65.7 (8.55)	0.116
Age range (years)	52-87	56-87	52-83	
Haematocrit (mean, (SD), n)	0.412 (0.044) 45	0.388 (0.035) 13	0.423 (0.044) 32	**0.00920**
MoCA score (range 0-30)	25.6 (3.45)	25.5 (3.50)	25.6 (3.48)	0.939
Education (years)	12.7 (3.21)	11.7 (3.46)	13.1 (3.07)	0.172
Time from stroke to consent (months)^1^	14.3 (12.0)	12.9 (8.83)	14.8 (13.1)	0.531
Modified Rankin score (range 0-6)	1 [1, 2]	1 [0, 2]	1 [0, 2]	0.873
NIH Stroke Scale (range 0-42)	1 [1, 2]	1 [0, 2]	1 [0, 2]	0.999
WMH volume (cm^3^)	14.6 [7.14, 32.1]	11.8 [6.78, 14.9]	15.3 [8.75, 32.8]	0.0822
Systolic Blood Pressure (mm Hg)	141 (13.9)	139 (16.5)	141 (12.9)	0.738
Diastolic Blood Pressure (mm Hg)	80.0 (9.19)	78.2 (10.4)	80.7 (8.74)	0.417

All data are reported as mean (SD), except modified Rankin score, NIH Stroke score and WMH volume which are reported as median [interquartile range]. Haematocrit data are given for visit #1 (available for 45 participants). Montreal Cognitive Assessment (MoCA) was used to detect cognitive impairment. Scoring in MoCA ranges from 0 to 30, with a score of 26 or higher indicating normal cognitive ability. These scores have been adjusted for educational level (+1 if the participant had 12 or more years of education). SBP and DBP data are the average over visit 1 and visit 2. P-values were derived from Student’s t tests, except for modified Rankin score, NIH Stroke scale score and WMH volume where Mann–Whitney tests were used.

Participants attended an initial screening visit (“visit 0”) and completed an eligibility check and gave informed consent. During the screening visit, education level and Montreal Cognitive Assessment (MoCA) scores were recorded (see Table 1). Following consent, participants attended two study visits (visit 1, visit 2) at least 7 days apart as specified in the study protocol [17]. At each study visit, participants underwent systolic/diastolic blood pressure (SBP/DBP) measurement, a cognitive test battery (see Supplementary file) and brain MRI scanning including pCASL. Participants then received either drug or placebo, according to the cross-over design, after which blood pressure, cognitive and MRI measurements were all repeated. All data reported here are from the set of measurements prior to treatment with drug or placebo.

Inclusion criteria were as follows. 1, radiological evidence of SVD, defined as: MRI evidence of lacunar infarct(s) ≤ 1.5 cm maximum diameter and/or confluent deep white matter leukoaraiosis (≥ grade 2 on the Fazekas scale). 2, Clinical evidence of cerebral small vessel disease defined as either: lacunar stroke syndrome with symptoms lasting >24 hours, occurring at least 6 months prior to visit 1; or: transient ischaemic attack lasting < 24 hours with limb weakness, hemi-sensory loss or dysarthria at least 6 months previously and with MR DWI performed acutely showing lacunar infarction. If MRI was not performed within 10 days of TIA, a lacunar infarction in an anatomically appropriate position as demonstrated on a subsequent MRI was also deemed eligible. 3, Age ≥ 50 years. 4, imaging of the carotid arteries with Doppler ultrasound, CT angiography or MR angiography in the previous 12 months, demonstrating < 70% stenosis in both internal carotid arteries or < 50% stenosis in both internal carotids if measured in previous 12-60 months.

Exclusion criteria included: known diagnosis of dementia; cortical infarction (>1.5 cm maximum diameter); systolic BP < 90 and/or diastolic BP < 50 mmHg; creatinine clearance <30ml/min; stroke or TIA within 6 months. For a full list of exclusion criteria see the published protocol [17].

### Blood Pressure measurement

SBP/DBP measurements were taken from each participant for visits 1 and 2, first on arrival after resting and then again after MRI scanning, using a validated Omron MX3 Plus machine.

### Magnetic Resonance Image Acquisition

Whole-brain perfusion MRI was acquired using a 3T scanner (Achieva TX MRI scanner, Philips Medical Systems, Eindhoven, Netherlands) at St George’s University Hospitals NHS Foundation Trust. Whole-brain T1-weighted, Fluid Attenuated Inversion Recovery (FLAIR) and pseudo-continuous arterial spin labelling (pCASL) images were acquired. All MRI data were acquired from brain scans performed on a Tuesday or Thursday between the hours of 10:00 and 12:00.

#### T1-weighted MRI

Whole-brain sagittal 3D T1-weighted images were acquired to enable tissue segmentation with the following protocol: Turbo Field Echo (TFE) sequence with an inversion pre-pulse, TFE factor 240 in multi-shot mode with 3000-ms shot interval, 8° flip angle, TR 7.9 ms, TE 3.8 ms, 1mm×1mm×1.5mm acquired resolution with interpolation to 1 mm isotropic resolution, 1 average and SENSE factor 2 for a 3-minute 47-second acquisition time.

#### FLAIR MRI

**Figure 1:**
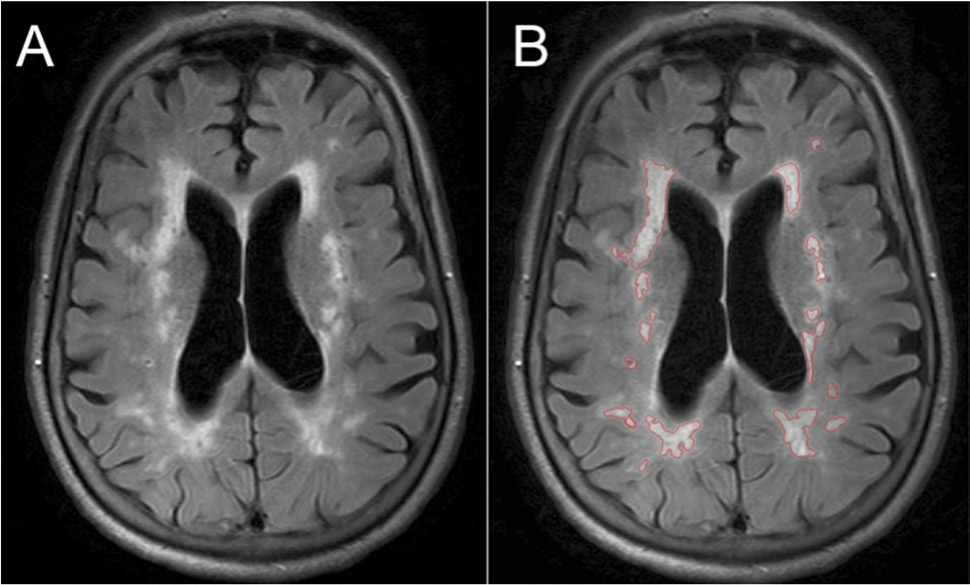
Example of white matter hyperintensities (WMH) in an older adult with small vessel disease. A, axial FLAIR scan, showing WMH. B, the same axial slice following semi-automated highlighting of WMH using Jim 7.0 software. Participant #022, female aged 77 y, with total WMH volume of 34,510 mm^3^ (across all scan slices).

**Figure 2:**
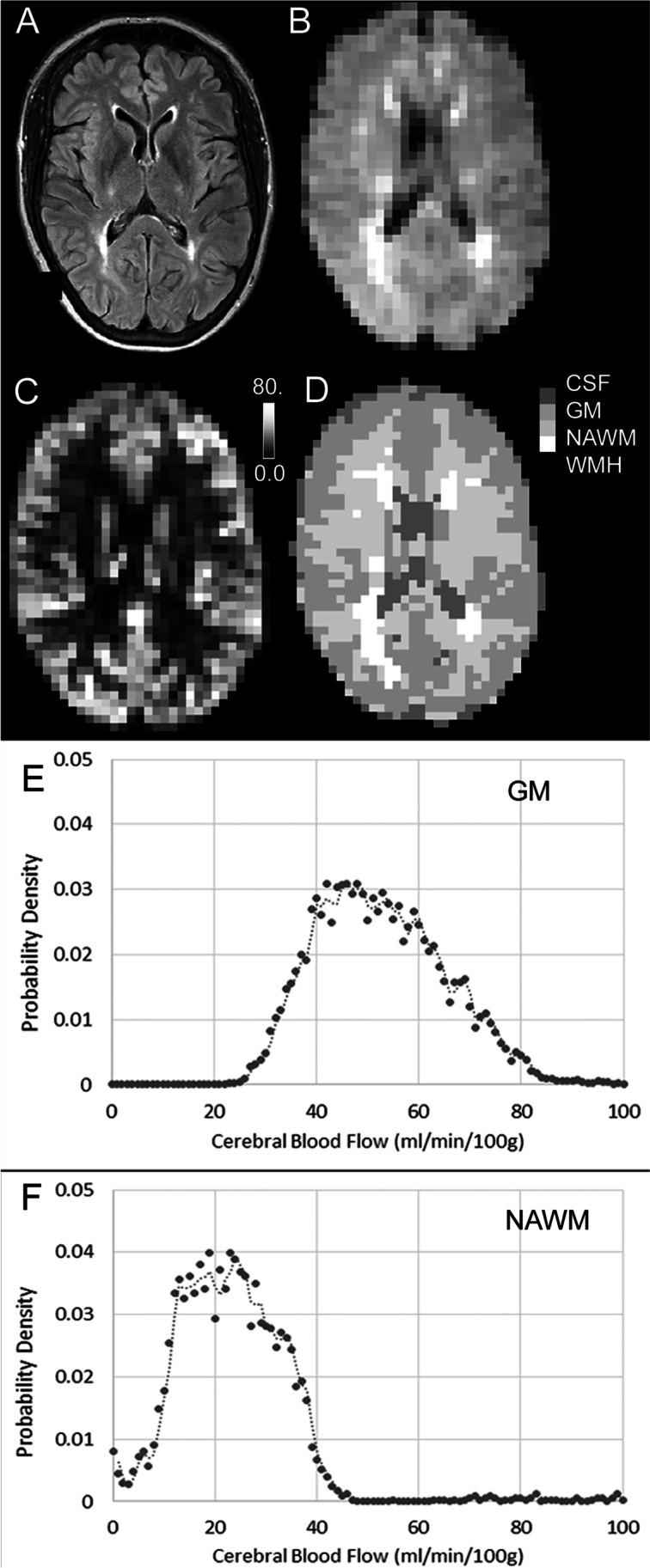
An example of regional anatomical and CBF mapping, with tissue segmentation. A, FLAIR image at full resolution. B, FLAIR image co-registered to the cerebral blood flow map, with voxels re-sized to be comparable with pCASL map. C, cerebral blood flow map, derived from pCASL. The calibration bar shows 0.0 - 80.0 ml/min/100g. D, tissue segmentation map for CBF computation. Each voxel has been defined as either: grey matter (GM), normal appearing white matter (NAWM), white matter hyperintensity (WMH) or cerebrospinal fluid (CSF). E, F: graphs show the probability density functions of cerebral blood flow values in voxels assigned as grey matter (in E) and normal appearing white matter (F). For this participant, median CBF in grey matter was 51.3 mm/min/100g and in NAWM 21.8 ml/min/100g. Participant #023, female, aged 56 y, visit 1.

2D T2-weighted axial FLAIR images were acquired to detect WMH using the following protocol: T2 weighted turbo-spin-echo sequences with selective fat suppression (TSE-SPIR), TR 11000 ms, TE 120 ms, TI 2800 ms, 0.65 mm×1.00 mm acquired resolution interpolated to 0.45×0.45 mm over 24-thick slices (5 mm thickness), with 2 averages and a 1.75 SENSE factor for a 3-minute 51-second acquisition time. Examples are shown in Figs. 1, 2.

#### pCASL MRI

Our pCASL protocol was developed based on the consensus recommendations of the ISMRM Perfusion study group and European consortium for ASL in dementia [15] using the Philips product pCASL sequence in the scanner 5.3 software release. A 64×64 acquisition matrix with 16 slices was used to acquire data with 4mm×4mm×7mm voxel size. Image readout was in 2D Echo-Planar Images (EPI).

Background tissue suppression was performed based on the ASL consensus recommendations [15]. Two inversion pulses were provided for background suppression as these represent an effective trade-off between tissue suppression and ASL signal. Background suppression pulses were applied at the beginning of the pulse sequence [15]. Spectral Pre-saturation with Inversion Recovery (SPIR) for fat suppression was applied to improve the contrast to noise of the blood perfusion signal. SPIR fat suppression did not add additional time to each slice acquisition. Pairwise acquisition of label and control images was performed. A total of 140 volumes (alternating with and without the spin labelling inversion pulse) were acquired in two separate 10-minute acquisitions using SENSE 2.3, and TE 8ms and TR 4300 ms with a labelling duration (τ) = 1800 ms and post-labelling delay (PLD) = 2000 ms. This was performed twice to increase ASL signal-to-noise ratio in the white matter [19]. This corresponds to a total acquisition time for the pCASL data of 20 min 6 s. A fixed labelling distance of 85 mm from the centre of the imaging block was used with the labelling slice positioned below the cerebellum at an angle perpendicular to the carotid arteries (visualized by time of flight angiography). Proton density-weighted images were acquired, to enable computation of CBF, using the pCASL sequence without the inversion pulse and background suppression, but with fat suppression and an increased TR 5000 ms to minimize T1 weighting (TE 9 ms with 8 averages). Proton density-weighted images were acquired in 40 s.

### Computation of CBF maps

The pCASL data acquisitions at each visit were corrected for subject movement using the FMRIB software library (FSL) function eddy_correct (https://fsl.fmrib.ox.ac.uk/FSL) [20]. An average pCASL map was then separately computed for each pCASL data acquisition. The average pCASL maps and the second proton density-weighted image were aligned to the initial proton density-weighted image in each scan session using the FSL Linear Image Registration Tool (flirt) [21]. These transformations were applied to the motion-corrected pCASL data to ensure all proton density-weighted and pCASL images were aligned in the same space. The aligned proton density-weighted images were averaged, and CBF was computed using *oxford_asl* (part of the FSL-BASIL toolset, https://fsl.fmrib.ox.ac.uk/fsl/fslwiki/BASIL) [22]. Cerebral blood flow in each voxel was calculated in physiological units of ml/min/100g using the standard equation for pCASL with (Equation 1) [15]:

1 $$\begin{array}{cc}CBF=\frac{6000\bullet \lambda \bullet \left({SI}_{control}-{SI}_{label}\right)\bullet \mathrm{exp}\left(\frac{PLD}{{T1}_{blood}}\right)}{2\bullet \alpha \bullet {T1}_{blood}\bullet {SI}_{PD}\bullet \left(1-exp\left(-\frac{\tau }{{T1}_{blood}}\right)\right)}& (\mathrm{in ml}/100\mathrm{g}/\mathrm{min})\end{array}$$

where SI_control_ and SI_label_ are the time-averaged signal intensities in the pCASL control and label images, respectively, and SI_PD_ is the signal intensity of a proton density-weighted image. Standard values were inserted into Equation 1 for the brain/blood partition coefficient, λ=0.9 ml/g, the labelling efficiency, α=0.85, longitudinal relaxation time of arterial blood T_1,blood_=1650 ms at 3T. An example pCASL map is shown in Fig 2C.

### White matter hyperintensity (WMH) delineation

WMHs were semi-automatically highlighted on each axial slice of the visit 1 FLAIR images (Fig. 1) using Jim 7.0 software (http://www.xinapse.com/jim-7-software/ Xinapse Systems Ltd, West Bergholt, Essex, UK). WMH were defined as hyperintense regions, which were (1) not due to the presence of blood vessels, and (2) not less than 10 mm^2^ in size, and (3) not a narrow band, one pixel wide, along the edge of the ventricles. A binary WMH image was generated, and the total WMH volume (in mm^3^) was computed for each participant. All WMH maps used here were produced by a single operator, blind to treatment allocation and to all clinical details (FAHH). A second, blinded operator (MMHP) also produced maps for a subset of participants (n=51), and interoperator agreement was good (intra-class correlation coefficient for total WMH volume ICC=0.855 [95% confidence interval: 0.760, 0.915], two-way random-effects model).

### Tissue Segmentation

For each scan session, T1-weighted images in native space were segmented into grey matter, white matter and cerebrospinal fluid (CSF) tissue probability maps, using a modified form of the standard Statistical Parametric Mapping (SPM) (SPM Version 12, https://www.fil.ion.ucl.ac.uk/spm/) geodesic shooting segmentation and normalisation procedure described in full in our previous papers [23, 24]. This procedure captures population-specific features, e.g. enlarged ventricles, and allows superior delineation of deep grey matter structures compared to the standard SPM pipeline. The binary WMH mask (co-registered into native T1-weighted space) was used to repair the tissue probability maps for misclassification caused by WMHs.

Native space T1-weighted and native space FLAIR were skull-stripped using FSL’s brain extraction tool (https://fsl.fmrib.ox.ac.uk/fsl/fslwiki/BET) [25] and co-registered to the average proton density-weighted image using boundary-based registration (FSL epi-reg) [26]. These 12 parameter linear transformations were used to align the corrected T1-weighted tissue probability maps and the binary WMH map to the CBF maps. A tissue mask in the average proton density-weighted image space was computed assigning each voxel to either grey matter, normal appearing white matter (NAWM), WMH or CSF, based on the maximum tissue probability.

### Computation of CBF in whole-brain tissue

For the alignment of the T1-weighted tissue segmentation images to the low-resolution pCASL images, it was necessary to apply a further segmentation step. This tissue segmentation procedure employs a novel application of a tissue segmentation algorithm to CBF maps [27]**.** It is designed to assign voxels with high CBF values to grey matter and low CBF values to white matter segments. The distribution of CBF values within the grey matter and white matter tissue masks computed in the Tissue Segmentation section (above) was entered as empirical priors to a hidden Markov random field model and segmentation (FMRIB's Automated Segmentation Tool, FAST) [27] to provide an improved segmentation of grey and white matter tissue from the CBF maps. This technique reduces the effects of partial volume and tissue classification errors at the boundary between grey and white matter tissue caused by the large pCASL image voxel size and the relative difference between voxel sizes of the native pCASL and T1-weighted images. In particular, this method assigns voxels with high CBF values at the grey/white matter tissue boundary to the grey matter segment and voxels with low CBF values at the grey/white matter tissue boundary to the white matter segment. To avoid misclassification of CSF and WMH regions, voxels in these regions were not entered into the FAST segmentation step. In our hands this approach was more successful than the more standard co-registration of tissue segmentations from high-resolution T1-weighted images to low-resolution pCASL images (not shown). For each participant at each scan session the median CBF values were calculated for total grey matter, NAWM and WMH. An example of tissue segmentation is shown in Fig. 2.

### Computation of CBF in deep grey matter structures

Cerebral deep grey matter structures were segmented on native space T1-weighted images using Freesurfer (Freesurfer Version 5.3.0, https://surfer.nmr.mgh.harvard.edu/fswiki/). The binary segmentations of the caudate, putamen and thalamus were aligned to the CBF maps by application of the affine transformation computed in Tissue Segmentation (above). Median CBF values were calculated for each of these three anatomical deep grey matter structures across the left and right cerebral hemispheres. An average of these three median values is reported for CBF in deep grey matter (DGM).

### Statistical Analysis

Statistical analyses were performed using SPSS (version 25.0). Unless otherwise stated data are presented as mean (SD). Test–retest reliability of CBF values (in ml/min/100g) between visit 1 and visit 2 was computed using intra-class correlation coefficients for whole brain, total grey matter, DGM, NAWM and WMH. Within-subjects coefficient of variation (wsCV) was also calculated for CBF values in each of these tissue types. Correlation of CBF values between tissue types was calculated using Pearson’s correlation coefficient. Bland-Altman plots were used to assess bias in CBF data between visit 1 and visit 2. Mean difference and upper and lower limits of agreement defined as ±1.96 standard deviations around the mean difference are reported. ANCOVA models were used to test for associations between: age (years), sex (M/F), blood pressure (SBP, DBP in mmHg) and CBF values. CBF was the dependent variable, sex was a fixed factor and age, SBP and DBP were co-variates. For CBF, SBP and DBP an average of the values for visit 1 and visit 2 was used in these analyses. No corrections were made for multiple comparisons, and p<0.05 was considered significant.

## Results

CBF maps were generated using pCASL in a cohort of older adults (age 66.9 (8.7) range: 52-87 years, N=54) all of whom had symptomatic SVD (Table 1, example in Figure 2). All participants had survived a lacunar stroke, and visit 1 occurred at least six months post-stroke. All participants completed visit 1 and visit 2 at least 7 days apart (mean (SD): 20 (19) days, range 7-117 days). Only four participants completed visit 2 more than 30 days after visit 1 (range 54-117 days). If these four participants were excluded, none of the parameters reported below changed significantly (P values in the range: 0.733 to 0.994; not shown).

For each participant CBF data were documented for visit 1 and visit 2 in whole brain and in four tissue types: grey matter (derived from all voxels defined as grey matter), DGM (from grey matter voxels within the caudate-putamen and thalami), NAWM and WMH. Average CBF values are given in Table 2.

**Table 2:** Test–retest reliability of CBF data across visit 1 and visit 2, at least 7 days apart.

	Visit 1 CBF Mean (SD)	Visit 2 CBF Mean (SD)	Visit 2 – Visit 1 CBF Mean (SD)^a^	ICC^b^ [95% C.I.]	wsCV (%)^c^	{wsCV}^2^ Mean (SD)^d^
Whole brain	25.45 (6.94)	27.00 (8.73)	1.55 (5.49)	0.758 [0.616, 0.852]	0.162 (16.2%)	0.0262 (0.0566)
Total grey matter	32.87 (7.71)	33.47 (8.82)	0.598 (4.67)	0.842 [0.743, 0.905]	0.123 (12.3%)	0.0152 (0.0255)
Deep grey matter	24.26 (6.19)	24.40 (6.94)	0.143 (4.48)	0.771 [0.636, 0.861]	0.146 (14.6%)	0.0213 (0.0282)
Normal appearing white matter	13.33 (4.75)	13.65 (4.98)	0.316 (2.46)	0.872 [0.790, 0.923]	0.168 (16.8%)	0.0282 (0.0557)
White matter hyperintensities.	9.185 (6.03)	9.75 (5.51)	0.561 (3.83)	0.780 [0.650, 0.866]	0.209 (20.9%)	0.0437 (0.0689)

^a^Mean difference between visit 2 value and visit 1 value. CBF data are given in units of ml/min/100g.

^b^Intra-class correlation coefficient. Single-measure, two-way random-effects model where both people effects and measures effects are random. Type A intra-class correlation coefficients using an absolute agreement definition. P<0.001 for all.

^c^ Within-subjects coefficient of variation (wsCV), cited as actual value and as a percentage.

^d^{wsCV}^2^ was computed and the mean and SD for this quantity is reported.

**Figure 3:**
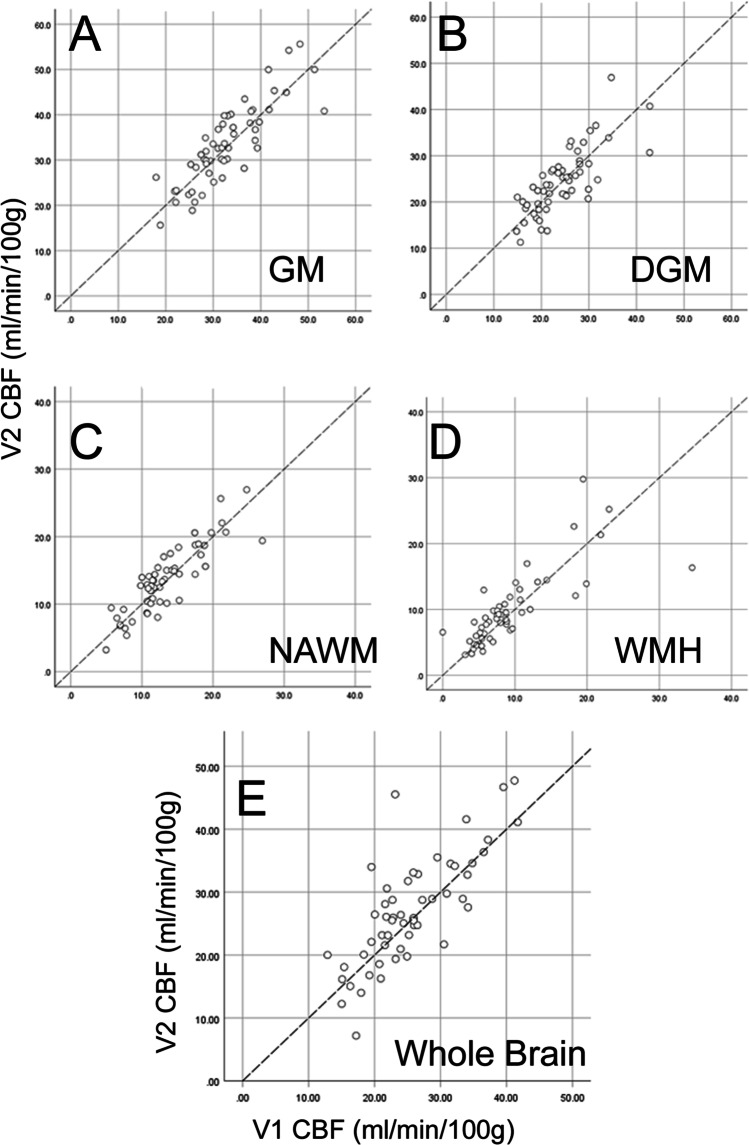
Test–retest reliability for CBF measurements (ml/min/100g) between visit 1 and visit 2. A) total grey matter, B) deep grey matter nuclei (caudate-putamen, thalamus), C) normal appearing white matter, D) white matter hyperintensities (WMH), E) whole brain. Each data point represents an individual participant, at study visit 1 (X-axis) relative to visit 2 (Y-axis). Dashed lines show the line of identity.

To explore internal consistency of the CBF measurement within participants, we compared CBF between visit 1 and visit 2. Scatter plots suggest good agreement between the two measurements (Figure 3). Bland-Altman plots further illustrate limits of agreement within CBF data between visits 1 and 2 (see supplementary Table S1, Figure S1). Intra-class correlation coefficients confirm high test–retest reliability for CBF in total grey matter and NAWM (Table 2) and reasonable test–retest reliability for whole brain, deep grey matter and WMH (Table 2).

CBF values were highly correlated between tissue types (Supplementary Table S2). There were positive correlations between total grey matter and deep grey matter, NAWM or WMH (R=0.924, 0.926, 0.642, respectively; p<0.001 for all, Table S2).

Comparing female participants with males, CBF was significantly higher in women in all tissue types (see Table 3, Figure S2). The difference was 5.9 ml/min/100g in grey matter, 4.3 ml/min/100g in DGM, 4.0 ml/min/100g in NAWM and 4.0 ml/min/100g in WMH (Table 3).

**Table 3:** A comparison of CBF between females and males.

	CBF (ml/min/100g)			
	Total N=54	Female n=15	Male n=39	Females vs Males, P-value
Total grey matter	33.2 (7.95)	37.4 (9.2)	31.5 (6.8)	**0.013**
Deep grey matter	24.3 (6.18)	27.4 (7.1)	23.1 (5.6)	**0.021**
Normal appearing white matter	13.5 (4.70)	16.4 (4.9)	12.4 (4.2)	**0.004**
WMH	9.47 (5.45)	12.4 (6.5)	8.4 (4.6)	**0.014**

CBF (ml/min/100g) data are presented as mean (SD) across participants, derived from the average of data from visit 1 and visit 2 for each participant. Student’s t tests were used to compare the CBF (ml/min/100g) values between females and males and between visit 1 and visit 2. WMH = white matter hyperintensities.

**Figure 4:**
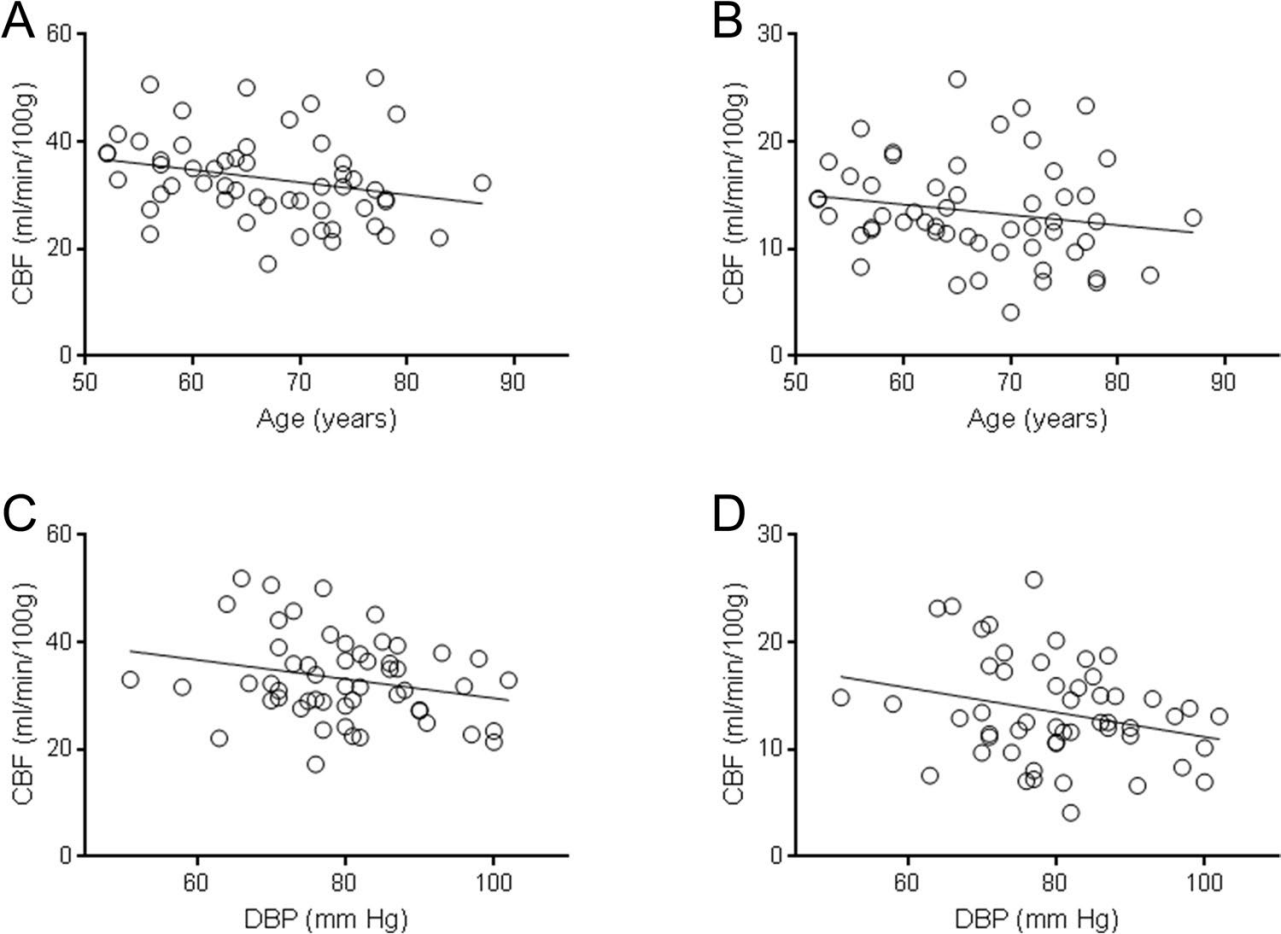
CBF with respect to age and diastolic blood pressure (DBP). CBF (ml/min/100g) for total grey matter (panels A, C) and normal appearing white matter (B, D) are plotted with respect to participant age (A, B) and DBP (C, D). CBF data are derived from the average across visit 1 and visit 2 for each participant (N=54). Solid lines show the least-squares linear best fit to the data.

ANCOVA models including sex, age and blood pressure (SBP and DBP) showed reasonable fit to the CBF data, albeit with a substantial amount of unexplained variance (R^2^ = 0.378 or less; p<0.001; see Table 4). Models indicated a significant decline in CBF in total grey matter, DGM and NAWM with increasing age (Table 4, Figure 4). Increasing DBP associated significantly with a decline in CBF in total grey matter, DGM and NAWM (Table 4, Figure 4). The models confirmed a significant association between female sex and higher CBF in all tissue types (Table 4).

**Table 4:** Relationship between CBF and age, blood pressure and sex, from ANCOVA models

	R^2^	Global Model P value	Age P value	SBP P value	DBP P value	Sex P value
Total grey matter CBF	0.378	**<0.001**	**0.005**	0.656	**0.007**	**0.003**
Deep grey matter CBF	0.299	**0.001**	**0.027**	0.568	**0.030**	**0.009**
NAWM CBF	0.363	**<0.001**	**0.016**	0.884	**0.007**	**0.001**
WMH CBF	0.275	**0.003**	0.137	0.063	0.638	**0.007**

The table shows R^2^ (unadjusted) for statistical model fit, and P values for ANCOVA with CBF were the dependent variable, sex (M/F) as a fixed factor and age, SBP and DBP as co-variates. For CBF, SBP and DBP an average of the values for visit 1 and visit 2 was used in these analyses. Abbreviations. CBF= cerebral blood flow; DBP = diastolic blood pressure; NAWM = normal appearing white matter; SBP = systolic blood pressure; WMH = white matter hyperintensities.

## Discussion

We have presented quantitative CBF maps in a well-characterized cohort of older persons with SVD. Test–retest reliability was high for total grey matter (which is dominated by cortical grey matter) and for NAWM, and reasonable for deep grey matter and WMH (Table 2). Based on these findings we consider pCASL a potentially useful tool to follow changes in CBF in older adults with SVD.

The CBF values reported (Table 2) are low relative to textbook values, which are derived from studies in healthy control subjects [28–34]. CBF values for grey matter are typically in the range 40-60 ml/min/100g for healthy younger adults [28–33]. Our CBF values are similar to data from healthy older people in some previous reports [7, 32, 33] and are lower than some others [30, 35, 36]. A recent longitudinal study of 468 older people reported average CBF across the age range 50-90 [37]. The average GM CBF at age 69 is reported as 38.2 ml/min/100g for women and 34.8 ml/min/100g for men [37], both of which are comparable with our values (37.4 ml/min/100g for women, 31.5 ml/min/100g for men, Table 2).

All our participants were older patients with symptomatic SVD, which may explain why our values are in the lower range. Our data are compatible with those reported previously for SVD populations (see a recent systematic review) [14]. In people with SVD, grey matter CBF was typically 20-46 ml/min/100g [7, 10, 12, 38, 39] (our measured value was 33 ml/min/100g). In those studies where white matter CBF was recorded, values were in the range 11-25 ml/min/100g [7, 10, 12, 18, 38, 39] (our value was 13 ml/min/100g).

In common with other ASL studies, owing to the large ASL voxel size, a potential confound in our data is the inclusion of some WM tissue in voxels classified as GM, and vice versa (termed the partial volume effect). Our technique for segmentation of CBF voxels does not provide an explicit partial volume correction within a voxel. This may lead to potential underestimates of grey matter CBF and overestimates of white matter CBF. The data for whole-brain CBF (Figure 3, Table 2) are not subject to this partial volume effect.

The ASL data in this study were all acquired with a single labelling delay, using methods derived from the ASL consensus recommendations [15]. No attempt was made to correct for variation between participants in terms of transit time or haemodynamic effects. This study was formulated to ensure high signal-to-noise ratios for CBF quantification in white matter. Consequently, there was a requirement to obtain sufficient perfusion signal within white matter, and an extensive, single post-labelling delay pCASL scanning protocol was adopted [17] based on methods for perfusion scanning in dementia [15]. The decision to acquire multiple averages with a single post-labelling delay (rather than multiple PLDs, with fewer averages at each PLD) may have led to underestimation of CBF. A recent study using simulations and some grey matter data, suggests that quantification of CBF is underestimated with a single PLD [40]. Despite this, our CBF measurements in human brain grey matter are comparable with those published by others using multiple post-labelling delays [32] or for pCASL using a single PLD [37]. Future studies would benefit from the improvement in accuracy and precision of CBF measurements provided by acquisition with multiple PLDs and quantification of labelling efficiency and blood T1.

Our CBF data for total grey matter and NAWM showed high test–retest reliability (Table 2, Figure 3). It is notable that NAWM had the highest intra-class correlation coefficient (Table 2). This suggests that even in NAWM, with low absolute CBF and low signal/noise ratio, our quantitation of CBF is robust. Reliability within DGM and WMH was reasonable (ICC: 0.771, 0.780, respectively) but lower than in total grey matter or NAWM, likely reflecting the smaller number of voxels sampled. Though our inclusion criteria permit a significant degree of large artery stenosis (up to 70%), this is unlikely to confound the test–retest reliability of CBF measurement within a given participant.

High test–retest reliability has previously been reported in healthy adult controls, comparing ASL-derived CBF measurements between scanners, investigators and time points [28, 29, 31, 36, 41]. In healthy controls (age 20-67 y) the change in CBF on re-scanning at least 6 months later was ± 25% in grey matter and ± 20% in white matter [28]. A recent study using a similar protocol to ours explored test–retest reliability in older people, with scans on average 42 days apart [42]. They reported similar ICC values to ours (0.84 for whole brain, 0.77 for WM) though only a minority of their cohort had significant SVD (8 out of 45, with severe subcortical WMH load) [42]. A study of pCASL in 40 healthy adults (age 18-65) found good consistency across 4 different scanners [29]. There was reasonable test–retest reliability between ASL measurements one week apart, with limits of agreement in grey matter of 25 - 45% (expressed as a fraction of the group average) across the four MRI scanners [29]. The limits of agreement in our data for older SVD patients were similar (28% for grey matter, 33% for NAWM, Table S1). Other groups have observed high test–retest reliability, comparing pCASL measurements less than 1 hour apart (correlation coefficients 0.93-0.96 in young controls, 0.82-0.93 in older controls) [33]. These values give an indication of the intrinsic variability in the measurement system. In light of these, the correlation coefficients we derived (ICC 0.842 for total grey matter, 0.872 for NAWM, Table 2) suggest low within-subject CBF variability over a timescale of 7 days in older adults with SVD.

We found significantly higher CBF in females than in males. This accords with many previous studies [28, 32, 43–48] (though not all [34]). This difference may in part reflect differences in circulating blood composition. The physiological range for haematocrit in pre-menopausal women is 10-15% lower than in men, with the gap narrowing above age 55. Female participants in our cohort had 9% lower haematocrit relative to male participants (Table 1), which may contribute to the observed difference in CBF. Other possible explanations include haemodynamic factors [34] and higher circulating levels of female sex hormones in women [45, 46].

In our statistical models, CBF declined with age as expected from previous reports [28, 30, 36, 44, 49, 50]. A large longitudinal study (309 healthy participants, age 20-89) [30] demonstrated a 30% decline in whole-brain CBF between 20 and 80 years of age, with significant association between declining CBF and cognitive impairment in older subjects [30]. In people with overt brain vascular disease, the decline in CBF with age may be more pronounced [11, 18].

In our models DBP had significant negative association with CBF (Table 4, Figure 4). Other groups have reported that hypertensive subjects have lower CBF relative to normotensive controls [51] and that high blood pressure, especially when uncontrolled, associates with declining CBF [52]. This may reflect chronic changes in the cerebral microvasculature of older people [53].

Our study has several limitations. First, the cohort is small (N=54). Though highly significant, our findings on test–retest reliability require validation in larger cohorts. Second, our cohort has an unequal sex distribution, with only 15 (28%) female participants. Even so, sex differences emerged that were highly significant and consistent with previous literature [45, 46]. Third, we did not attempt to validate our pCASL data within subjects against a second CBF measurement modality (either SPECT, PET or DSC-MRI) [16].

*In conclusion*, we report quantitative CBF mapping using pCASL in a clinically relevant older population with symptomatic SVD. Test–retest data from our study and others [18, 29, 30, 33] suggest that pCASL is well tolerated and may be a technique that can contribute to clinical practice. This method may be applicable for detecting group differences (as endpoints, for comparing interventions) or within-subject changes in longitudinal studies of disease progression. We found higher CBF in women than in men, in all tissue types studied. This highlights the importance of sex-matching in trials with CBF as an endpoint. Our data suggest that pCASL sequences are a robust tool for CBF measurement in clinical and research settings.

## Availability of data and material (data transparency)

The data supporting this report are available from the corresponding author upon reasonable request.

## Supplementary Information

Below is the link to the electronic supplementary material.

Supplementary file1 (DOCX 292 KB)

## Data Availability

Not applicable.
