# Supplementary material for: Test–retest reliability of arterial spin labelling for cerebral blood flow in older adults with small vessel disease
Source: Transl Stroke Res. 2022 Jan 26;13(4):583–94. doi: 10.1007/s12975-021-00983-5 (PMC9232403; doi:10.1007/s12975-021-00983-5)
Supplement: Supplementary file 1 — Supplementary file1 (DOCX 292 KB) [file 12975_2021_983_MOESM1_ESM.docx]

**Supplementary Material**

**Test-retest reliability of arterial spin labelling for cerebral blood flow in older adults with small vessel disease**

Lauren R Binnie^1*^, Mathilde MH Pauls^1,2*^, Philip Benjamin PhD FRCR^1,5^, Mohani-Preet K Dhillon MRes^1^, Shai Betteridge^3^, Brian Clarke^2^, Fearghal AH Hainsworth^1^, Franklyn A Howe^1^, Usman Khan^2^, Christina Kruuse^6^, Jeremy B Madigan^5^, Barry Moynihan^2,10^, Bhavini Patel^2^, Anthony C Pereira^2^, Egill Rostrup^9^, Anan BY Shtaya PhD FRCS^1^, Catherine A Spilling PhD^1^, Sarah Trippier^7^, Rebecca Williams^7^, Jeremy D Isaacs^1,2^, Thomas R Barrick^1^ and Atticus H Hainsworth^1,2,**^

*^1^Molecular & Clinical Sciences Research Institute, St George’s University of London, UK, ^2^Department of Neurology, St George’s University Hospitals NHS Foundation Trust London, ^3^Department of Neuropsychology, St George’s University Hospitals NHS Foundation Trust London, ^5^Department of Neuroradiology, St George’s University Hospitals NHS Foundation Trust London, ^6^Department of Neurology and Neurovascular Research Unit, Herlev Gentofte Hospital, Denmark, ^7^South London Stroke Research Network, ^9^Mental Health Centre, Glostrup, University of Copenhagen, Glostrup, Denmark, ^10^Dept of Medicine, Royal College of Surgeons in Ireland, Beaumont Hospital, Dublin, Ireland.*

* These authors contributed equally to this work.

**corresponding author: Dr AH Hainsworth PhD, St George's University of London, Cranmer Terrace, London SW17 0RE, United Kingdom. tel +44 208 725 5586 fax +44 208 725 2950. Email: [ahainsworth@sgul.ac.uk](mailto:ahainsworth@sgul.ac.uk). ORCID: orcid.org/0000-0001-7877-8013

**Supplementary Methods**

Study population

All data are from a cohort of older adults with radiological and clinical evidence of symptomatic SVD (N=54, demographic details in Table 1). These participants were all recruited as part of a double-blinded, placebo-controlled, phase-II clinical trial, Perfusion by Arterial spin labelling following Single dose Tadalafil In Small vessel disease (PASTIS; European Union Clinical Trials Register number 2015-001235-20, registered 13/05/2015) [1]. The trial received ethical approval from the UK National Research Ethics Service (REC reference: 15/LO/0714). Written informed consent was obtained from all participants or their next of kin. Participants were enrolled by members of the trial team and randomised to order of treatment (tadalafil 20 mg, placebo; oral administration). The randomisation list was generated in advance by Sharp Clinical Services, Crickhowell, Powys, UK.

The trial commenced 4th September 2015. Participants were recruited from St George’s Hospital and local Participant Identification Centres. All visits, data management and trial coordination were performed at the St George’s site. The trial ended when the pre-determined recruitment target was met (25 January 2018). This trial was a cross-over design so all participants consented to receive the trial drug, tadalafil, though all measurements reported here were made prior to dosing. Participants were recruited from St George’s University Hospital NHS Foundation Trust and local Participant Identification Centre sites between 14 September 2015 and 25 January 2018. All patient visits, data management and trial coordination were performed at St George’s Hospital.

Participants attended an initial screening visit (“visit 0”), and completed an eligibility check and gave informed consent. During the screening visit, education level and Montreal Cognitive Assessment (MoCA) scores were recorded (see Table 1). Following consent, participants attended two study visits (visit 1, visit 2) at least 7 days apart as specified in the study protocol [1]. Participants were asked to refrain from alcoholic drinks for 48 hours prior to each visit or grapefruit containing products. They were also asked to refrain from xanthine containing drinks or foods (coffee, coca cola, tea, chocolate) or smoking prior to each visit.

At each study visit, participants underwent systolic/diastolic blood pressure (SBP/DBP) measurement, a cognitive test battery (listed below) and brain MRI scanning including pCASL. Participants then received either drug or placebo, according to the cross-over design, after which blood pressure, cognitive and MRI measurements were all repeated. All data reported here are from the set of measurements prior to treatment with drug or placebo.

Inclusion criteria were as follows. 1, radiological evidence of SVD, defined as: MRI evidence of lacunar infarct(s) ≤ 1.5 cm maximum diameter and/or confluent deep white matter leukoaraiosis (≥ grade 2 on the Fazekas scale). 2, Clinical evidence of cerebral small vessel disease defined as either: lacunar stroke syndrome with symptoms lasting >24 hours, occurring at least 6 months prior to visit 1; or: transient ischaemic attack lasting < 24 hours with limb weakness, hemi-sensory loss or dysarthria at least 6 months previously and with MR DWI performed acutely showing lacunar infarction. If MRI was not performed within 10 days of TIA, a lacunar infarction in an anatomically appropriate position as demonstrated on a subsequent MRI was also deemed eligible. 3, Age ≥ 50 years. 4, imaging of the carotid arteries with Doppler ultrasound, CT angiography or MR angiography in the previous 12 months, demonstrating < 70% stenosis in both internal carotid arteries or < 50% stenosis in both internal carotids if measured in previous 12-60 months.

Exclusion criteria included: known diagnosis of dementia; cortical infarction (>1.5 cm maximum diameter); systolic BP < 90 and/or diastolic BP < 50 mmHg; creatinine clearance <30ml/min; stroke or TIA within 6 months. For a full list of exclusion criteria see the published protocol [1].

Neuropsychological testing

Each participant completed a 15-minute battery of neuropsychological tests at visit #1 and visit #2. These are listed below.

- Reaction Time (RTI) subtest of the CANTAB battery
- Speed of Information Processing (SoIP) subtest of the Brain Injury Rehabilitation Trust Memory and Information Processing Battery (BMIPB)
- Digit Span (DS) Forwards & DS-Backwards, subtest of the Repeatable Battery for the Assessment of Neuropsychological Status (RBANS)
- Semantic Fluency, subtest of RBANS

**Supplementary Figure S1.** Bland-Altman plots show the average of CBF values on visit 1 and visit 2 (X-axis) relative to the difference between CBF for visit 1 compared to visit 2 (visit 2 value – visit 1 value, Y-axis). Units are ml/min/100g. Plots are shown for total grey matter, deep grey matter, normal appearing white matter and white matter hyperintensities (WMH). Each data point represents an individual participant (N=54). Horizontal solid line shows the average difference between visits. Limits of agreement are shown as dashed lines (±1.96 SD around the mean difference).

**Supplementary Figure S2**. Comparison of CBF in males and females. A) total grey matter. B) deep grey matter (DGM). C) normal appearing white matter. D) white matter hyperintensities (WMH). Data from 39 male and 15 female participants. Box-whiskers show the median, inter-quartile range and 90% confidence interval. Open circles show outliers with values 1.5 - 3 box lengths from the upper or lower quartile. Asterisks (*) show outliers with a value more than 3 times the interquartile range from a quartile.

**Supplementary Table S1.** Mean difference and limits of agreement (LOA) for regional CBF across visit 1 and visit 2 calculated using Bland-Altman plots. N=54.

|  | Mean difference, (visit 2 – visit 1) | Lower LOA | Upper LOA |
| --- | --- | --- | --- |
| Total grey matter | 0.598 | -8.546 | 9.741 |
| Deep grey matter | 0.143 | -8.634 | 8.92 |
| Normal appearing white matter | 0.316 | -4.515 | 5.146 |
| White Matter hyperintensities | 0.561 | -6.937 | 8.059 |

Data shown are for CBF (ml/min/100g).

**Supplementary Table S2.** Pearson correlation coefficients of CBF measurements across tissue types

|  | Deep GM | NAWM | WMH |
| --- | --- | --- | --- |
| Total GM | 0.924  P<0.001 | 0.926  P<0.001 | 0.642  P<0.001 |
| Deep GM |  | 0.847  P<0.001 | 0.531  P<0.001 |
| NAWM |  |  | 0.668  P<0.001 |

| Data are shown for visit 1 data. Similar results were obtained for visit 2 data (not shown). |
| --- |

**Supplementary References**

1. Pauls MMH, Clarke N, Trippier S, Betteridge S, Howe FA, Khan U, Kruuse C, Madigan JB, Moynihan B, Pereira AC, Rolfe D, Rostrup E, Haig CE, Barrick TR, Isaacs JD, Hainsworth AH. Perfusion by Arterial Spin labelling following Single dose Tadalafil In Small vessel disease (PASTIS): study protocol for a randomised controlled trial. Trials. 2017; 18.
